# Where do students in the health professions want to work?

**DOI:** 10.1186/1478-4491-7-74

**Published:** 2009-08-18

**Authors:** Deborah Schofield, Susan Fletcher, Jeffery Fuller, Hudson Birden, Sue Page

**Affiliations:** 1 Northern Rivers University Department of Rural Health, School of Public Health, Faculty of Medicine, University of Sydney, Lismore, Australia

## Abstract

**Background:**

Rural and remote areas of Australia are facing serious health workforce shortages. While a number of schemes have been developed to improve recruitment to and retention of the rural health workforce, they will be effective only if appropriately targeted. This study examines the factors that most encourage students attending rural clinical placements to work in rural Australia, and the regions they prefer.

**Methods:**

The Careers in Rural Health Tracking Survey was used to examine the factors that most influence medical, nursing and allied health students' preference for practice locations and the locations preferred.

**Results:**

Students showed a preference for working in large urban centres within one year, but would consider moving to a more rural location later in life. Only 10% of students surveyed said they would never work in a rural community with a population of less than 10 000. Almost half the sample (45%) reported wanting to work overseas within five years. The type of work available in rural areas was found to be the factor most likely to encourage students to practice rurally, followed by career opportunities and challenge

**Conclusion:**

The decision to practise rurally is the result of a complex interaction between a number of factors including ethnicity, discipline, age and sex, among others. Incentives that aim to entice all students to rural practice while considering only one of these variables are likely to be inadequate.

## Background

Australia is a large country with a dispersed population of about 21 million persons. Limited access to health services remains a significant problem for people living in rural and remote areas of Australia [1-4], due to difficulties in attracting and retaining health professionals to rural practice [5-9]. Various schemes have been devised by the Australian government to improve the recruitment and retention of the rural health workforce, such as the introduction of scholarships, increasing the number of students with rural background, and financial incentives [1-3,5,10-14]. However, to be most successful they need to target students most likely to work in such areas [3].

The current literature has identified a number of factors that can influence clinicians in making location decisions. Students from a rural background are more likely to work in rural areas than those from urban backgrounds [1,3-6,15-21], with the likelihood increasing if the practitioner's spouse is also from a rural background [1,17,19]. Males are reportedly more likely than females to work in rural and remote regions [1,5,22]. Students with undergraduate or postgraduate training in rural areas are more likely to return to rural practice than those without [1,3-5,15,17,20,23,24]. Studies in a number of Australian states have also demonstrated that undertaking rural placements during the course of their study increases the probability of subsequent rural practice and that longer placements may increase rural practice outcomes [25-27].

There have been more studies on practice location preference for doctors than for other health professionals. These studies concluded that doctors typically choose to work in rural areas for lifestyle reasons [3,19]. Health science students reported that they are influenced to choose rural practice by a combination of factors, including personal and professional needs and social context [11], with these factors also noted for a focus group of nutrition and dietetic students and recent graduates [16].

The current study aims to add substantially to previous research by answering the questions "What factors most influence medical, nursing and allied health students' preference for practice location?" and "What are their locations of preference?" This study is unique in that it includes all health disciplines and takes account of family factors that affect employment preferences. In addition, it is specific about practice location and asks about the time frame for preferred practice locations.

## Methods

The Careers in Rural Health Tracking Survey (CIRHTS) was designed to examine employment preferences and decision criteria that medical, nursing and allied health students use in determining whether to practise in a rural, remote or urban setting.

The survey questions students in detail on many aspects of their lives, and because the decision to move to a rural area may also involve a partner or spouse, most items have space for both student and spouse responses. The five components of the survey are:

1. personal characteristics and family relationships (nine items)

2. education and employment (six items)

3. location preferences (five items)

4. rural background (four items)

5. influencing factors (one item covering 18 factors).

Ethics approval was received from the University of Sydney Human Research Ethics Committee in 2006. Further details on the survey have been published in a previous paper [28]. Data analysis was undertaken using SAS 9.1 (SAS Institute, Cary, North Carolina, United States of America).

## Results and Discussion

In the first year of CIRHTS, 243 students were invited to complete the survey while on rural placement in northern New South Wales (NSW). These students came from 11 different universities predominantly from the east coast of Australia. The duration of the placements varied in length from several weeks to several months. Students varied from first to final year of their undergraduate clinical studies, with the exception of some medical students, some of whose courses are by graduate entry.

Of the 243 students, 121 agreed to participate: a 50% response rate. The study included 43 medical students (36%), 46 nursing students (38%) and 32 allied health students (26%). Allied health disciplines included pharmacy, occupational therapy, physiotherapy, dietetics, speech therapy, social work and podiatry. The ages of the students ranged from 20 to 33 years for medical students, 19 to 47 years for allied health students and 18 to 56 years for nursing students. Foreign ancestry (students identifying themselves as being of any ancestry other than Australian/New Zealand) was more common among medical students than among allied health or nursing students. The nursing cohort, being older, was more likely to be married or in a de facto relationship. A summary of student demographics is provided in Table 1.

**Table 1:** Description of the student sample

	**Number**	**Average age**	**Female**	**Married** **(de facto relationship)**	**Have children**	**Foreign ancestry**	Rural background*
Medicine	43	25.2	22 (51%)	6 (14%)	0 (0%)	22 (51%)	10 (23%)

Nursing	46	27.8	38 (83%)	13 (28%)	15 (33%)	8 (17%)	41 (89%)

Allied health	32	25.5	18 (56%)	3 (9%)	4 (13%)	9 (28%)	20 (63%)

*Rural background was defined as town of population less than 100 000.

### Rural background

Forty-eight students (40%) reported coming from a capital city or major urban centre (population > 100 000); 20% came from a regional city or large town (25 000 – 100 000). Fewer students (17%) came from a small town (10 000 – 25 000), while 23% came from a small rural community (< 10 000). There was a relatively larger proportion of students from regional or rural backgrounds than might be expected based on the distribution of the Australian population, partly due to rural students' self-selecting rural placements and some rural students having enrolment or scholarship obligations to undertake training in a rural area.

**Figure 1:**
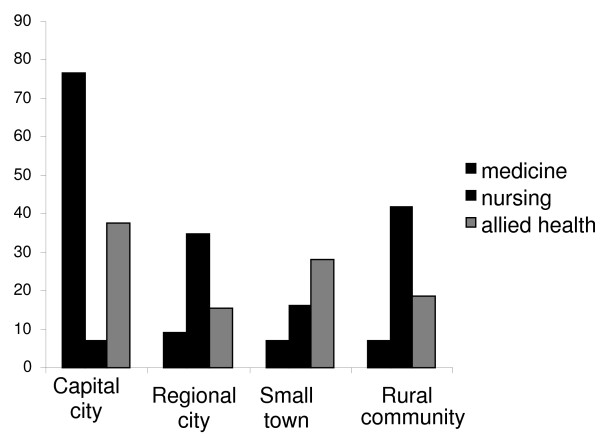
**Student background by discipline**.

Allied health and medical students were more likely than nurses to have a capital city or major urban centre background (Fig. 1).

Some 89% of the sample attended high school in Australia, with 60% of them spending at least one year in a school outside a capital city or major urban centre.

### Location preferences

**Figure 2:**
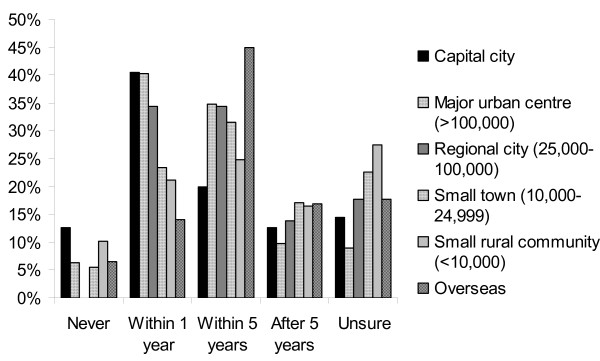
**Student location preferences**.

Figure 2 indicates that 40% of students want to work in larger centres within one year, while small rural communities appear to be more attractive than cities in the longer term (17% compared to 13% for capital cities, in 5+ years). Few students indicated that they would never want to work in a rural area (10%). A large number of students were unsure about working rurally (23% in a small town and 28% in a rural community). Nearly half the students (45%) were looking to work overseas within the next five years. (It should be noted that students could select each period more than once, e.g. happy to work in a capital city, major urban centre or overseas in the next year. Therefore, percentages within each category along the x-axis do not add to 100. Percentages within each location do, however.)

Students aged 30 or over were more likely to never want to work in a capital city (p < 0.05). No students aged less than 21 would never want to work in a small town or rural community within Australia. Participants in their twenties preferred to work in larger centres (>25 000) within a year, but were open to work in more rural locations later in life.

Background appeared to play a major role in location decisions, with students showing a preference for working in a town size they were familiar with. One quarter of those from a rural background never wanted to work in a capital city, while another 38% were unsure about doing so. On the other hand, the majority (57%) of students with a capital city background wanted to work in a city within one year, and around one third were unsure about working outside an urban area.

Reflecting the background differences between disciplines, nursing students were most likely to never want to work in a capital city, or were unsure about doing so, while 28% wanted to work in a rural community of fewer than 10 000 people within one year. Medical students, on the other hand, were unsure about working outside urban centres. This is most likely a demographic effect rather than a disciplinary effect, with about three quarters of the medical cohort reporting being from a capital city and about half reporting foreign ancestry (migrants tend to gravitate to Australian cities). One third of allied health students wanted to work overseas within a year, with another 46% wanting to leave Australia within five years: a total of 80% of young allied health professionals.

Students of Asian descent were more likely to never want to work in a town of fewer than 10,000 people than those of either Australian or European ethnicity. Over half of the European and Asian students in the sample wanted to work in a capital city within one year, compared to 38% of Australians. However, Europeans were also more likely never to want a city-based job, with 24% saying they would never work in a capital city, compared to 15% of Australians and just 5% of Asian students. Ancestry reflects the background students most identify with and may not necessarily reflect where the student was born.

**Figure 3:**
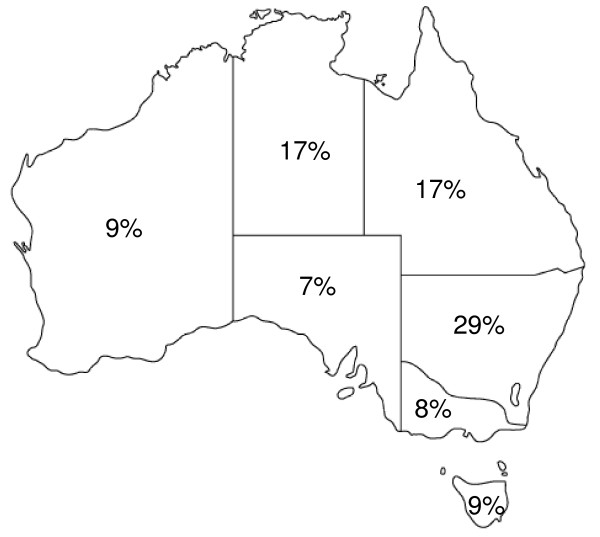
**Average proportion of students wanting to work in rural Australia, by state**.

The cities of Brisbane and Melbourne were the most attractive to students, with 62% and 58% of respondents saying they would be happy to work in each city, respectively. Perth also fared well, with 49% of students prepared to work in the western capital. Around a third of the students were happy to work in the other capital cities of Adelaide, Hobart, Darwin and Canberra. Few students indicated they would like to work in rural regions of states other than New South Wales (Fig. 3). Worst-off was the northern region of South Australia, an area composed mostly of desert, where only 2% of students would be happy to work. Other desert areas fared better: 17% of students said they would work in the Northern Territory or the Kimberly region of Western Australia (WA). On the other hand, just 4% of students were willing to work in the south-eastern corner of WA.

**Figure 4:**
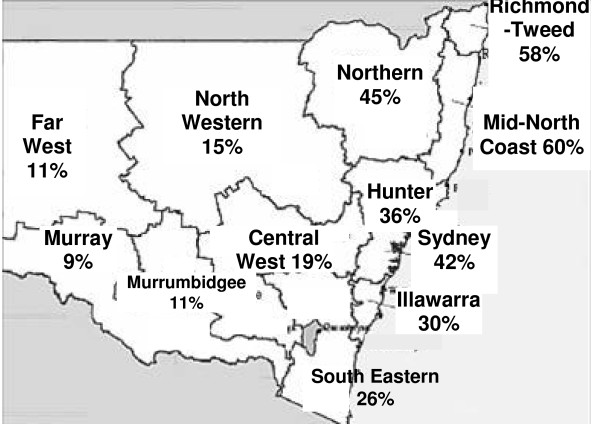
**Proportion of students wanting to work in each region of New South Wales**.

Within NSW, the north coast area was the most popular, with fewer students wanting to work inland (Fig. 4). Despite this finding, only 38% of students said living close to the coast was somewhat or very important to them.

Preferences for future practice location were influenced by the desire to undertake postgraduate studies for just over half (56%) of the sample.

### Influencing factors

The type of work available was the factor most likely to influence the location preferences of students, with 51% of the sample saying this would strongly encourage them to work rurally and a further 23% reporting that it would slightly encourage them to do so. Only two students indicated that work type would discourage them from working in a rural area. Career opportunities and challenges were also found to be strong encouragers of rural practice (Table 2).

**Table 2:** Factors most likely to encourage students to work rurally

		**Top influencing factor**	**% saying "encourage"**	**% saying "strongly encourage"**
**Age**	<21	Career opportunities	33%	33%
	
	21–24	Type of work	18%	53%
	
	25–29	Cost of accommodation Type of work	21% 21%	65% 65%
	
	30+	Environment for raising children	0%	60%

**Sex**	Males	Cost of living	38%	48%

	Females	Type of work	28%	41%

**Children**	Yes	Environment for raising children	0%	83%
	
	No	Type of work	25%	48%

**Discipline**	Medicine	Cost of accommodation Challenge	28% 39%	44% 39%
	
	Nursing	Type of work	6%	56%
	
	Allied health	Career opportunities	56%	38%

**Regional background**	Population >100 000	Type of work Cost of accommodation Cost of living	33% 19% 29%	43% 48% 43%
	
	25 000–100 000	Challenge	42%	42%
	
	10 000–24 999	Type of work	0%	83%
	
	<10 000	Rural lifestyle/culture	30%	40%

**Ethnicity**	Australian/NZ	Type of work	15%	57%
	
	Asian	Challenge	33%	50%
	
	European	Cost of accommodation	0%	75%

While their rural placement was not generally noted as the most important reason influencing where they would prefer to work, 65% of students reported their NRUDRH placement as a factor that would encourage them to work in a rural area, with 50% of students also reporting a previous rural placement as being a positive factor. Only one student reported the NRUDRH placement and only two students another rural placement as a negative factor.

A number of students provided other reasons for wanting to work in a rural area, including professional support, coastal location, happiness, owning a farm, loving the bush and wanting to live there, financial incentives and work flexibility.

Factors that were less likely to encourage students to work rurally were typically those that applied to only a minority of students, and so were most often given a neutral rating, including access to children (custodial arrangements) and bonding or other contracting work requirements. Apart from these factors, students were most discouraged from rural practice by a lack of public transport. Students of European ancestry appeared to be discouraged by the lifestyle and culture of rural areas.

## Conclusion

CIRHTS indicates that the decision of health professionals to work in a rural location is not determined simply by background (although consistent with other studies, rural background was important [1,3-6,15-21]), but varies between individuals and indeed locations as a result of the complex interaction of many factors.

The choice is not simply between "rural" and "urban" locations. Under the umbrella of the term "rural" there are many different areas, each of which is attractive (or not) to different people for different reasons. Studies that distinguish only between rural and urban, or between rural, remote and urban, are likely to under- or overestimate the likely shortages in certain areas.

The results of this study support those of numerous previous researchers who report a positive relationship between rural placements and later rural practice [1,3-5,15,17,20,23-27]. Of the students surveyed, only one reported that the NRUDRH placement would discourage him or her from working rurally in the future. With students almost unanimously finding their rural placement to encourage rural practice, further Government investment in rural placement initiatives would appear to be worthwhile. Adams et al. noted four factors positively associated with rural employment perceptions after a rural placement: (1) friendliness and support in rural areas; (2) isolation and socialization problems associated with living and working in rural areas; (3) enjoyable aspects of living in a rural area; and (4) opportunities that working in a rural area provides, such as work autonomy and the variety of skills used [11]. Shoo et al. noted that students with a city background chose rural practice for the beauty of the outdoors, to be close to the coast or to be close to family, while those who stayed in the city cited lack of rural exposure, graduate programme opportunities and being close to home [25].

This study demonstrated that student intentions about practice location over the life course and in the short term may differ from their longer-term plans. For older respondents and those with children, a good environment for raising children was an important factor encouraging rural practice. Students may seek specific professional and/or life experiences before settling into a practice location. An unexpected and somewhat alarming finding of the current study was the high proportion of medical, nursing and particularly allied health students looking to work overseas in the next five years. However, it may be possible to design incentives that capitalize on this desire for travel. Programmes whereby employees can work for four years at reduced pay and take their fifth year as paid leave are becoming more common in sectors such as finance and education, and the introduction of similar programmes for health professionals should be considered, particularly in rural areas.

Factors within two categories were consistently nominated as those most likely to influence student location decisions: "career factors" (e.g. type of work, career opportunities and challenges) and "financial factors" (e.g. cost of accommodation and cost of living). As these two categories appear to be the most important to students when deciding where to work, emphasizing them when designing initiatives to encourage rural practice should result in greater effectiveness. The variety of work that rural doctors undertake and the skill development that results is likely to interest females, allied health students and those from a city background, for example, as career factors are highly important to these students. A focus on the cost of living, on the other hand, may be especially useful in attracting males, medical students, Europeans and students in their late 20 s.

Given the large proportion of students who are unsure about working rurally, appropriately targeted incentives have the potential to significantly improve rural health workforce numbers by persuading these students to give rural practice a try. The Australian Government has recently released a report summarizing the existing incentives; most of these are for medical practitioners, a small number for nurses and none for allied health, with the exception of one mental health programme and the University Departments of Rural Health. There is a clear need for further incentives for allied health professionals to work in rural areas [29].

## Competing interests

The authors declare that they have no competing interests.

## Authors' contributions

DS led the study; DS and SP conceived the original study; all authors were members of the project team and worked on survey design; SF was the project officer who implemented the survey and undertook the analysis. All authors read and approved the final manuscript.
